# Heart rate variability and hematological parameters in pregnant women

**DOI:** 10.1002/jcla.23250

**Published:** 2020-02-24

**Authors:** Ahmad I. Al‐Shafei, Shaza M. Musa, Duria A. Rayis, Mohamed F. Lutfi, Ola A. El‐Gendy, Ishag Adam

**Affiliations:** ^1^ Unaizah College of Medicine Qassim University Unaizah Saudi Arabia; ^2^ Faculty of medicine Najran University Saudi Arabia; ^3^ Faculty of Medicine University of Khartoum Khartoum Sudan; ^4^ College of Medicine Qassim University Qassim Saudi Arabia; ^5^ Department of Obstetrics and Gynecology Unaizah College of Medicine Qassim University Unaizah Saudi Arabia

**Keywords:** cardiac autonomic modulations, heart rate variability, hemoglobin, mean platelets volume, red cell distribution width, white blood cells

## Abstract

**Background:**

There are few researches on hematological parameters (hemoglobin, red cell distribution width [RDW], white blood cells [WBCs], mean platelets volume [MPV], and heart rate variability [HRV]). There are no published data on this concept (HRV and hematological parameters) during pregnancy.

**Methods:**

A cross‐sectional study was conducted at Saad Abul Ela hospital in Khartoum, Sudan during the period of July to August 2018. Pregnant women with singleton, a live baby, were enrolled in this study. Clinical history and examination were performed. HRV (autonomic modulation) was assessed using time and frequency domain HRV indices.

**Results:**

One hundred and five pregnant women were enrolled. The median (quartile) of the age, parity, and gestational age was 30.0 (25.0‐35.0) years, 1.0 (0‐3.0), and 38.0 (32.0‐39.0) weeks, respectively. While there were positive correlations between hemoglobin and low frequency (LF), RDW and high frequency (HF), WBCs and HF Norm, WBCs and LF/HF, MPV and HF Norm, LF Norm and LF/HF, there was no significant correlation between the hematological (hemoglobin, WBCs, RDW, and MPV) and HRV parameters. Linear regression analysis showed no significant association between age, parity, gestational age, body mass index, hemoglobin, RDW, and HRV variables. The Log_10_ WBCs were negatively associated with Log_10_ HF (ms^2^/Hz). MPV was positively associated with LF Norm and negatively associated with HF Norm.

**Conclusion:**

The study failed to show significant associations between age, parity, gestational age, hemoglobin, RDW, and HRV variables. The WBCs were negatively associated with HF. MPV was positively associated with LF Norm, and it was negatively associated with HF Norm.

**Abbreviations**
BMI: body mass index
HF: high frequency
HRV: heart rate variability
LF: low frequency
MPV: mean platelets volume
MPV: mean platelets volume
RDW: red cell distribution width
TP: total power
VLF: very low frequency
WBCs: white blood cells

## 1 INTRODUCTION

Heart rate variability (HRV) is an informative tool in assessing cardiac autonomic modulations; it has a prognostic value in cardiac diseases, and it is widely used to assess the impact of autonomic imbalance on various diseases such as type 2 diabetes mellitus and obesity,^1^, ^2^ coronary heart disease, 3 and inflammatory conditions.^4^ Previous studies have shown altered sympathovagal balance (autonomic modulations) during pregnancy and labor.^5^, ^6^, ^7^, ^8^, ^9^ There are previous publications of blood parameters, for example, hemoglobin,^10^ iron deficiency anemia (IDA),^11^ red cell distribution width (RDW),^12^ mean platelets volume (MPV),^13^ folate and vitamin B_12_ deficiencies,^14^ and white blood cells (WBCs).^15^, ^16^ However, there are no published data on this concept (HRV and hematological parameters) during pregnancy. Previously, performing complete blood count (CBC) was a hectic procedure and its results might not be accurate; however, now, CBC can be performed using the automated procedures within few minutes and reasonable cost.^17^

We have recently observed a high level of anemia (57.7%), an IDA (12.1%) folate and vitamin B_12_ deficiency among pregnant Sudanese women.^18^, ^19^ Moreover, we reported different HRV and autonomic modulations in pregnant Sudanese women.^20^, ^21^ The current study was conducted to investigate the associations between HRV, autonomic modulations, and hematological parameters (hemoglobin, WBCs, RDW, and MPV) in normal pregnant Sudanese women.

## 2 METHODS

A cross‐sectional study was conducted at Saad Abul Ela tertiary maternity hospital in Khartoum, Sudan during the period of July – August 2018.

An inclusion criterion was any Sudanese pregnant women with singleton, a live baby. Participants who were in labor had any disease, such as hypertension, diabetes, heart disease or any other medical condition complicating pregnancy, or taking any medication (except supplements) were excluded.

After signing an informed consent form each woman, all the women were examined and personal, medical, and obstetrics history were taken. Then, weight and height measurements were taken, and body mass index (BMI) was computed using the equation [BMI = weight in Kg/(height in meters)^2^].

The details of the HRV recordings were mentioned in our previous work.^20^, ^21^ In summary, after taking a complete bed rest for 10 minutes, systolic and diastolic blood pressures were recorded using sphygmomanometer. Then, a 5 minutes HRV recording was obtained using biocom 3000 recorder (heart rhythm scanner—version 2.0—biocom technologies) as recently described.^2^ HRV and the autonomic modulation of the heart were examined using both time and frequency domains analysis. HRV parameters include the following: the NN intervals (SDNN), the square root of the mean squared differences of successive NN intervals (RMSSD), total power (TP), very low frequency (VLF), low frequency (LF), and high frequency (HF). Meanwhile, the sympathetic and parasympathetic autonomic modulations were evaluated via normalized low frequency (LF Norm) and high frequency (HF Norm), respectively.

Because HRV has a circadian rhythm,^22^ all measurements of HRV were done in the morning between 8 and 12 AM

Then, 2 mL of blood was withdrawn from every participant in an ethylene diamine tetra acetic acid and analyzed for a complete blood count using an automated hematology analyzer and following the manufacturers' instructions (Sysmex KX‐21) previously described.^17^, ^23^

Guided by Bujang and Baharum findings,^24^ a sample size of 105 pregnant women was calculated to give the significant minimum difference in the correlations (*r *= .27) between HRV and hematological parameters. The calculated sample size was set to give the study 80% power and a difference of 5% at *α* = .05.

### 2.1 Statistics

Data were entered in computer using SPSS (version 20.0) software (SPSS Inc). The Shapiro‐Wilk test was used to assess if the continuous variables were normally distributed. Mean (SD) and median (interquartile) were used to express the normally and non‐normally distributed, respectively. Data (hematological and HRV parameters) which were not normally distributed were log‐transformed to allow using the parametric statistical tests. The Pearson's correlation coefficients were used to assess the associations between two continuous variables. Stepwise multivariate linear regression analyses were performed where (Log) HRV parameters (one by one) as the dependent variable and the other clinical and hematological parameters were the independent variables. Log‐transformed variables were entered when appropriate. A two‐sided *P*‐value <.05 was considered statistically significant.

## 3 RESULTS

One hundred and five pregnant women were enrolled in the study. The clinical and obstetrical characteristics were shown in Table 1. The median (quartile) of the age, parity, and gestational age was 30.0 (25.0‐35.0) years, 1.0 (0‐3.0), and 38.0 (32.0‐39.0) weeks, respectively. Hemoglobin and MPV were the only normally distributed variables, and their mean (SD) was 11.7 (1.0) g/dL and 9.9 (1.3) fL, respectively.

**Table 1:** The clinical and obstetrical characteristics of pregnant Sudanese women

Variables	Median	Quartiles
Age, years	30.0	25.0‐35.0
Parity	1.0	0‐3.0
Gestational age, weeks	38.0	32.0‐39.0
Body mass index, kg/m^2^	30.0	25.1‐33.9
Systolic blood pressure, mm/Hg	120.0	110.0‐120.0
Diastolic blood pressure, mm/Hg	80.0	70.0‐80.0
Mean heart rate, beat/minute	95.5	88.3‐103.8
Hemoglobin^a^, g/dL	11.7	1.0
RDW, %	14.1	13.4‐15.0
WBC (×10^3^,mm^3^)	8.1	5.8‐10.0
MPV^a^, fL	9.9	1.3

a Mean (SD).

The Pearson's correlation showed no significant correlations between the clinical, hematological parameters, and VLF. With exception of a significant positive association between gestational age and LF, none of the clinical variables (age, gestational age, parity, and BMI) was correlated with the HRV parameters. While there were positive correlations between hemoglobin and LF, RDW and HF, WBCs and HF Norm, WBCs and LF/HF, MPV and HF Norm, LF Norm and LF/HF, there was no significant correlation between the hematological (hemoglobin, WBCs, RDW, and MPV) and HRV parameters, Table 2.

**Table 2:** Correlation between clinical, hematological, and heart rate variability in pregnant Sudanese women

Variables	VLF (ms^2^/Hz)		LF(ms^2^/Hz)		HF(ms^2^/Hz)		LF Norm(nu)		HF Norm(nu)		LF/ HF	
	*r*	*P*	*r*	*P*	*r*	*P*	*r*	*P*	*r*	*P*	*r*	*P*
Age, year	−.109	.268	.005	.958	.053	.0592	−.052	.598	.031	.753	.039	.696
Parity	.054	.586	.061	.539	.007	.944	.001	.991	.04	.887	.004	.995
Gestational age, weeks	.072	.467	.225	.021	.112	.256	.080	.418	−.085	.391	.089	.367
Body mass index,(kg)/ (m)^2^	.004	.969	.092	.353	.030	.761	.120	.221	−.117	.236	.111	.259
Hemoglobin, g/dL	.172	.079	.199	.042	.138	.162	.033	.741	.008	.936	.005	.959
RDW, %	.125	.341	.137	.298	.268	.038	−.199	.126	.223	.087	−.214	.100
WBC (×10^3^, mm^3^)	.083	.105	.091	. 105	.003	.105	.060	. 105	−.204	.037	.202	.039
MPV, fL	−.093	.481	.059	.654	−.126	.336	.320	.013	−.354	.005	.352	.006

Linear regression analysis showed no significant association between age, parity, gestational age, BMI, hemoglobin, RDW, and HRV variables. The Log_10_ WBCs were negatively associated with Log_10_ HF (ms^2^/Hz) (−1.261, *P* = .036). MPV was positively associated with LF Norm (5.373 fL, P .026), and it was negatively associated with HF Norm (−6.828 fL, *P* = .005), Table 3 and Table 4.

**Table 3:** Factors associated with LgVLF, LgLF (ms^2^/Hz), and Lg HF in pregnant women using linear regression analyses

Log of the variable	Lg VLF (ms^2^/Hz)			LgLF(ms^2^/Hz)			LgHF(ms^2^/Hz)		
	Coefficient	SE	*P*	Coefficient	SE	*P*	Coefficient	SE	*P*
Age, year	−0.995	1.032	.342	0.166	1.154	.886	0.858	1.082	.434
Parity	−0.005	0.244	.985	−0.230	0.273	.405	−0.177	0.256	.496
Gestational age, weeks	0.587	1.747	.739	2.260	1.953	.256	2.793	1.833	.138
Body mass index, (kg)/(m)^2^	0.785	1.185	.513	0.209	1.325	.875	−0.891	1.243	.479
Hemoglobin, g/dL^a^	0.076	0.078	.338	0.110	0.087	.217	0.058	0.082	.485
RDW, %	1.062	1.527	.492	0.334	1.708	.846	2.225	1.603	.175
WBC (×10^3^, mm^3^)	−0.732	0.547	.191	−0.576	0.612	.354	−1.261	0.574	.036
MPV, fL^a^	−0.025	0.054	.638	0.023	0.060	.698	−0.074	0.056	.195
Mean heart rate	−0.094	0.017	<.001	─ 0.065	0.018	<.001	−0.002	0.014	.897

Abbreviatiuon: SE, standard error.

a Normally distributed variables and the log were not used.

**Table 4:** Factors associated with LF Norm, HF Norm, and Lg LF/HF in pregnant women using linear regression analyses

Variable	LF Norm (nu)			HF Norm (nu)			Lg LF/HF		
	Coefficient	SE	*P*‐value	Coefficient	SE	*P*‐value	Coefficient	SE	*P*‐value
Age, year	−38.382	44.345	.393	29.322	43.763	.508	−0.691	0.899	.448
Parity	−0.710	10.500	.947	−0.666	10.362	.949	−0.054	0.213	.802
Gestational age, weeks	−29.045	75.093	.702	21.922	74.106	.769	−0.533	1.522	.729
BMI,(kg)/(m)^2^	68.123	50.944	.191	−61.040	50.275	.234	1.100	1.033	.295
Hemoglobin, g/dL	2.519	3.361	.459	−1.822	3.317	.587	0.052	0.068	.449
RDW, %	−72.548	65.661	.278	81.432	64.798	.218	−1.891	1.331	.165
WBC (×10^3^, mm^3^)	31.820	23.513	.186	−37.101	23.204	.120	0.685	0.477	.161
MPV, fL	5.373	2.300	.026	−6.828	2.270	.005	0.098	0.047	.044
Mean heart rate									

Abbreviatiuon: SE, standard error.

Adjusted for mean heart rate.

## 4 DISCUSSION

Our results showed a significant association between HRV and WBCs (Log_10_WBCs was negatively associated with Log_10_ HF). A similar finding demonstrating an association between HRV and WBCs was recently reported.^15^ Independent relationships of 24‐hours HRV (measured by SDNN) with WBCs were recently reported in a large population‐based study.^16^ WBCs which are marker for a low‐grade inflammatory process were observed to be an independent predictor of cardiovascular disease.^25^ The association between HRV and WBCs could be explained by epinephrine (the main sympathetic neurotransmitter), which was reported to be negatively associated with HRV and positively associated with inflammatory parameters.^26^ Moreover, previous researches reported expression of adrenoceptors on immune cells.^27^, ^28^ A medical induced sympathectomy was reported to reduce inflammatory process.^29^ An anti‐inflammatory process seems to be associated with vagal activity.^30^

In the current study, MPV was positively associated with LF Norm and was negatively associated with HF Norm. Our finding (MPV was positively associated with LF) points to increased cardiac sympathetic modulations in association with MPV. A significant association between MPV and increased sympathetic activity was previously reported in patients with vasovagal syncope.^13^ An increased MPV (which are large‐sized metabolically and enzymatically more active compared with the normal‐sized platelets) was associated with increased cardiovascular risk factor.^31^ The reported prognostic role of increased MPV in patients with acute myocardial infarction could be explained by increased sympathetic activity and decreased HR variability.^32^ Peripheral activation, splenic release of platelets, and increased thrombocytopoiesis might explain the impact of sympathetic activity on MPV.^33^ High adrenaline concentration can cause changes in the shape and size of platelets through adrenoreceptor activation and thereby, influence MPV.^32^ Nearly one third of platelets sequestered in the spleen through the activation of sympathetic system.^34^ However, to our knowledge, there is no published work investigating the effect of increased sympathetic activity on platelet turnover during pregnancy.

In the current study, hemoglobin and RDW were not associated with HRV. Previous studies have reported significant associations between hemoglobin,^10^ IDA,^11^ RDW,^12^ and HRV. To the best of our knowledge, this is first work of homological parameters and HRV. However, the hematological values such as hemoglobin, RBC, WBCs, and platelets are expected to change due to increase plasma volume during pregnancy expansion.^35^, ^36^ Moreover, it is worth to be mentioned that HRV parameters during pregnancy are shifted toward sympathetic dominance.^5^

## 5 CONCLUSION

The study failed to show significant associations between age, parity, gestational age, BMI, hemoglobin, RDW, and HRV variables. The WBCs negatively associated with HF. MPV was positively associated with LF Norm and negatively associated with HF Norm.

### 5.1 Limitations of the study

There was no case of severe anemia in this study. Other markers for inflammation such high C‐reactive protein were not investigated. HRV is a good method, but it is not the only one to the analysis of cardiovascular autonomic control. Further research taking into account the development of other approaches such as chronotropic competence indices in graded exercise testing and baroreflex sensitivity to the analysis of cardiovascular autonomic control is needed.^37^, ^38^

## CONFLICT OF INTERESTS

The authors declare that they have no competing interests.

## ETHICS APPROVAL

The study received ethical approval from the Research Board at the Faculty of Medicine, Alneelain University, Sudan. The reference number is 2016/09. Written informed consent was obtained from all the enrolled patients.
